# From Studio to Rehab: A Debilitating Form of Anti-HMGCR Myopathy

**DOI:** 10.7759/cureus.40825

**Published:** 2023-06-22

**Authors:** Comfort Anim-Koranteng, Okeoghene Akpoigbe, Michael Miller, Yelena Averbukh

**Affiliations:** 1 Internal Medicine, Harlem Hospital Center, New York, USA; 2 Department of Pathology and Cell Biology, Columbia University Irving Medical Center, New York, USA; 3 Medicine, St John's Riverside Hospital, New York, USA

**Keywords:** autoimmune, atorvastatin, antibodies to signal recognition particle (anti-srp), antibodies to 3-hydroxy-3-methyl-glutaryl-coenzyme a reductase(anti-hmgcr), statin-induced myopathy, myositis

## Abstract

Immune-mediated necrotizing myopathy is a subtype of immune-mediated myopathy associated with or without statin use. Statins, or HMG-CoA reductase inhibitors, are the most prescribed medications for dyslipidemia. The statin-associated myopathic syndromes range from asymptomatic elevations in creatine kinase to severe debilitating muscle weakness with associated rhabdomyolysis and elevated liver enzymes. Clinical improvement occurs upon discontinuation of statins, but some patients do not recover completely. Diagnostic tests include electromyography, muscle biopsy, myositis autoantibody panel, and antibodies against the HMGCR. Here, we present a case of anti-HMGCR-related myopathy associated with atorvastatin.

## Introduction

Immune-mediated myopathies are rare diseases, of which immune-mediated necrotizing myopathy (IMNM) is a subtype. Its prevalence ranges from 9 to 14 cases per 100,000 people in a Sweden population-based study [1] and 7-11 per 100,000 people in the United States [2]. The incidence of IMNM among patients on statins accounts for 2-3 cases in 100,000 patients with a median age of 64 years [2].

Anti-signal recognition particle (SRP) myopathy, HMGCR myopathy, and antibody-negative IMNM are the three recognized serological subtypes of IMNM. Most patients with IMNM have either anti-SRP or anti-HMGCR myopathy, with only 10% being antibody-negative, so-called seronegative IMNM [1]. Anti-HMGCR myopathy is the most common autoimmune myopathy associated with statin exposure, especially atorvastatin, compared to simvastatin or rosuvastatin [2].

Statin-induced myopathy does not affect most patients on statin therapy, highlighting the role of other risk factors in the pathogenesis of the disease. Potential associated risk factors for statin-induced myopathy include patient-specific risk factors such as age, medical co-morbidities, genetic makeup, and other medication uses [3]. In addition, environmental risk factors may play a role. Still, elucidating those factors may be complicated, given the similar prevalence of anti-HMGCR myopathy in countries where statins are used daily.

Most patients with IMNM usually present with fatigue, myalgia, muscle weakness, and tendon pain, all of which worsen with exercise and with a preference for the proximal muscle groups. The onset time of statin-induced myopathy varies, with a mean onset time of six months after initiation of statin therapy [4].

## Case presentation

A 63-year-old man with a medical history of type 2 diabetes mellitus, hypertension, and asthma presented with a one-day history of immobilization after a fall. A month prior, the patient endorsed progressive bilateral leg weakness. His medication before hospitalization includes metformin, lisinopril, amlodipine, atorvastatin, cyclobenzaprine, and albuterol. He was hemodynamically stable and afebrile, with physical examination findings significant for proximal muscle weakness, intact sensation, and hyporeflexia in all extremities.

Laboratory tests demonstrated elevated creatine kinase (CK) levels of 16,000 U/l (reference range of 39-308 U/l) and elevated transaminases of five times the upper limit of normal (ULN).

The cervical and lumbar spine MRI was negative for cord compression or any space-occupying lesion. The MRI showed diffuse short tau inversion recovery (STIR) hyperintense signal abnormality throughout the paravertebral musculature indicative of muscle edema and inflammation. With concern for myopathy, atorvastatin was withheld, and the left lateralis muscle biopsy showed mild atrophy and scattered necrosis of the myofiber and regeneration without evidence of vasculitis or amyloidosis. The myositis panel was normal with negative anti-SRP, but he tested positive for HMG Co-A antibodies (IgG >200 units, ULN of 19 units).

The patient was initially started on high-dose intravenous steroids. Once thiopurine s-methyltransferase enzyme activity was evaluated as normal, he was also initiated on azathioprine therapy. Intravenous immunoglobulin (IVIG) was considered but deemed contraindicated in this patient, given the simultaneous presence of pulmonary embolism (PE) since thrombotic complications are known to be associated with IVIG therapy and could increase the clot burden.

The patient’s clinical course was complicated by dysphagia, respiratory failure, and PE of the right main pulmonary artery, confirmed with computed tomography pulmonary angiogram. He was given iv rituximab therapy, percutaneous enterogastric tube insertion for nutritional requirements, intubated for mechanical ventilation, and anticoagulated with apixaban. However, he was successfully extubated before discharge. HyperCKemia was managed with intravenous fluids, and the CK gradually improved from 16,000 to 5,000. On a follow-up visit, steroids had been tapered off, and the patient was only on azathioprine with regular physical and respiratory therapy without recovery of muscle strength.

## Discussion

Myopathy is a known adverse side effect of statin therapy. The presentation varies from asymptomatic, mild symptoms like fatigue and myalgia to severe forms like hyperCKemia and necrotizing autoimmune myopathy with associated complications [5]. CK, a marker of muscle breakdown with an elevation of more than ten times its ULN, is termed severe myopathy. Rhabdomyolysis is diagnosed with CK levels at least five times the ULN but ranges from approximately 1,500 to over 100,000 units [5].

Patients with IMNM demonstrate progressive proximal muscle weakness, elevated CK of 10-100 times the ULN, necrosis found on muscle biopsy, and poor recovery with withholding statins, as the case discussed. In addition, dysphagia and respiratory insufficiency are more frequent in groups of patients with positive anti-SRP antibodies than in those with anti-HMGCR antibodies [6].

**Figure 1:**
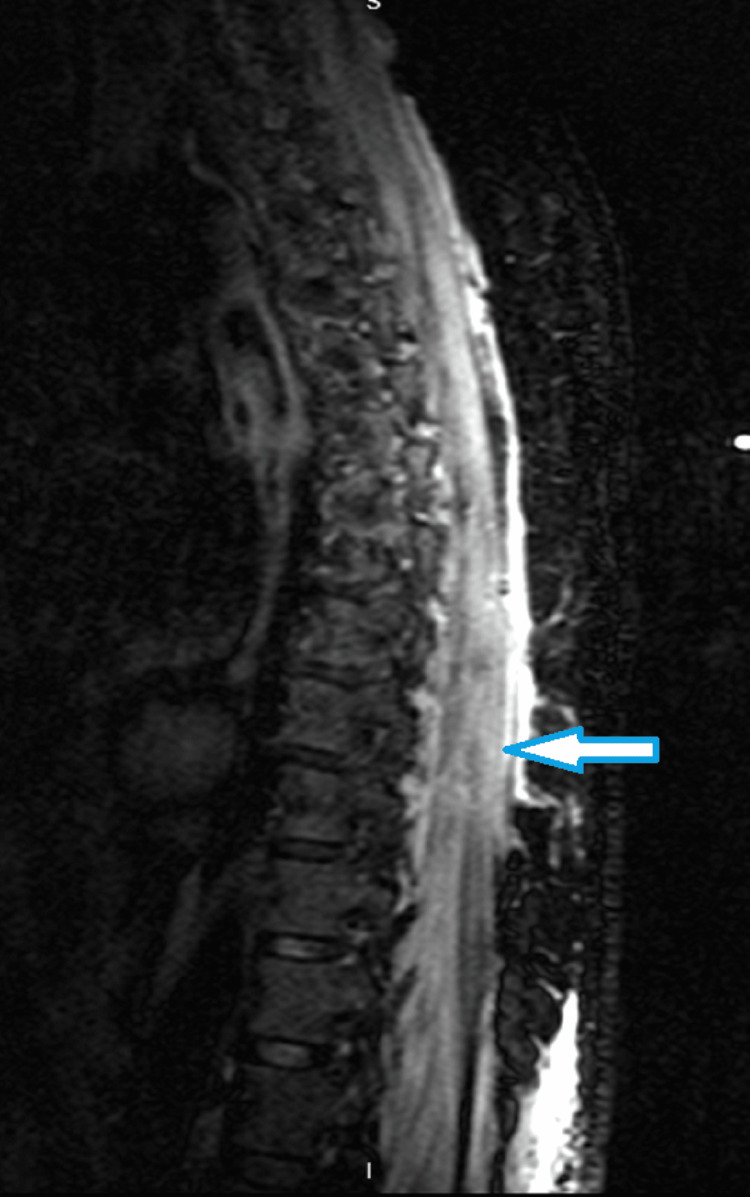
MRI of the thoracic spine, sagittal view Diffuse T2/STIR hyperdensities seen in paravertebral and thoracic musculature (arrow shows a part of the hyperdensity)

Diagnostic tests for IMNM include electromyography (EMG), muscle biopsy, antibody test, and sometimes MRI of the muscle to guide the biopsy site and assess disease severity and prognosis [2]. A bedside test of EMG with readily available results demonstrates low amplitude, short-duration motor-unit potentials with increased spontaneous activity [2,7]. MRI of the thoracic spine (Figure 1) shows T1 weighted and STIR sequence hyperintensities of the paravertebral and shoulder musculature, which reflect active inflammation and a suitable parameter of disease improvement when resolution occurs [2,8].

**Figure 2:**
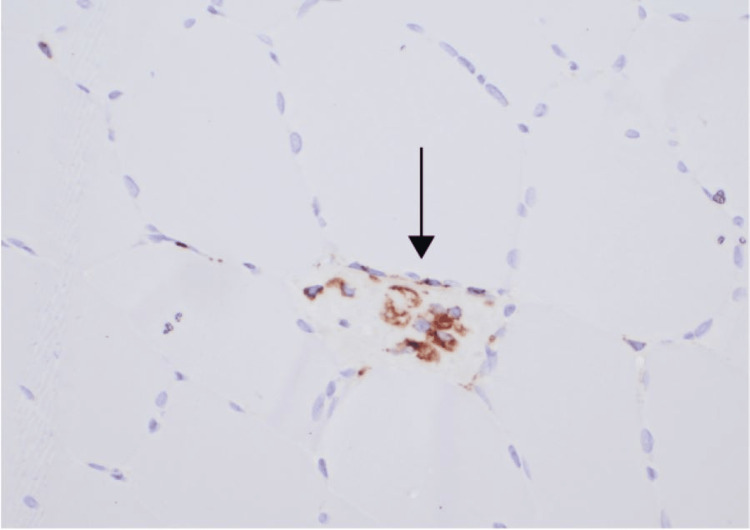
Immunostaining of muscle fiber CD68 immunostaining highlights myophagocytosis (arrow) of muscle fiber

**Figure 3:**
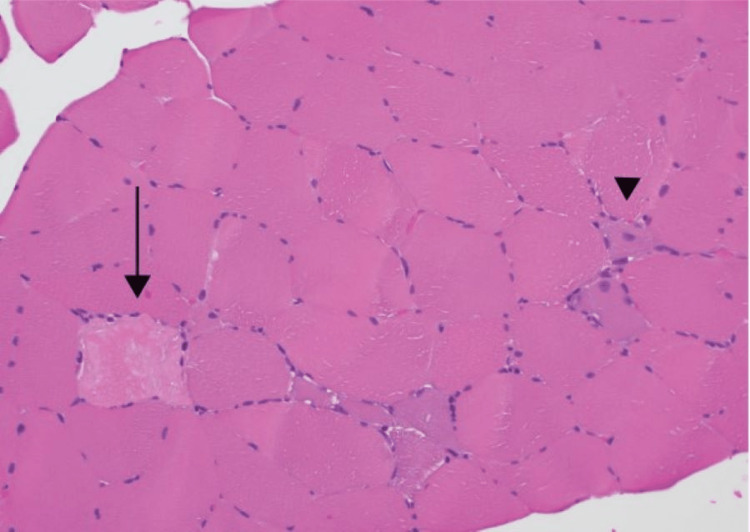
Muscle biopsy histopathology Scattered throughout the fascicles, individual necrotic (arrow) and basophilic regenerative myofibers (arrowhead) are identified.

Cell necrosis and regeneration are the most prominent histologic features in muscle biopsy specimens from patients with IMNM, with similar findings demonstrated in the case presented (Figures 2, 3).

Major histocompatibility complex (MHC) class-1 and membrane attacking complex (MAC) stain by 3,3'-Diaminobenzidine immunohistochemical were negative. MHC and MAC are seen in inflammatory myopathy like dermatomyositis and polymyositis but not as strongly in IMNM [9] with inconclusive sensitivity and specificity. Thus, IMNM diagnosis is based on the holistic involvement of clinical, histopathological, and serological features.

Autoantibodies against HMG-CoA reductase are predominant in patients with necrotizing myopathy with statin exposure despite occurrence among statin-naive patients [10].

Patient-based management of IMNM involves a multidisciplinary team with the goals of statin cessation and avoidance for life, immunosuppressive therapy, and rehabilitation.

The resolution of myopathy varies with the withdrawal of statins. Those with severe anti-HMGCR myopathy require monotherapy or a combination of corticosteroids, IVIG, and steroid-sparing immunosuppressants (SSIs) [11]. Mycophenolate mofetil, azathioprine, and methotrexate are the commonly used SSIs [12]. Azathioprine is the preferred agent with a 3 g/kg/body weight dose. Therapy for refractory patients is rituximab; others are cyclosporine, cyclophosphamide, and etanercept.

The case discussed started with intravenous methylprednisolone, then azathioprine, and rituximab. Unfortunately, he was not a candidate for IVIG due to the morbidity of PE. Therefore, the prognosis of the disease is patient based. A single-center-based study in the United States identified patients with anti-HMGCR myopathy with mild forms who did not require aggressive immunosuppressants and presented with late-onset weakness. In contrast, patients with anti-SRP myopathy have the severest form with early-onset weakness, prominent oropharyngeal dysphagia, and irreversible muscle damage, contrasting with the case presented [12,13].

## Conclusions

In summary, recovery of patients ranges from a complete resolution of weakness to those with persistent disability despite intense immunosuppressant treatment. There is an attribute of good prognoses, such as recovery with early treatment with IVIG; however, the percentage of IMNM patients with persistent weakness despite intense immunosuppressant treatment is growing significantly, as is the case of our patient who progressed from an artist in a studio to a resident of a rehabilitation center. This calls for further research to determine what other factors significantly influence the severity of symptoms and recovery.
